# Vitamin D Status in Women with a History of Infertility and Decreased Fecundability: A Population-Based Study

**DOI:** 10.3390/nu15112522

**Published:** 2023-05-29

**Authors:** Johanna Lumme, Laure Morin-Papunen, Paula Pesonen, Sylvain Sebert, Elina Hyppönen, Marjo-Riitta Järvelin, Karl-Heinz Herzig, Marja Ojaniemi, Maarit Niinimäki

**Affiliations:** 1 Research Unit of Clinical Medicine, University of Oulu, 90014 Oulu, Finland; laure.morin-papunen@oulu.fi (L.M.-P.); marja.ojaniemi@oulu.fi (M.O.); maarit.niinimaki@oulu.fi (M.N.); 2 Medical Research Center, Oulu University Hospital and University of Oulu, 90220 Oulu, Finland; karl-heinz.herzig@oulu.fi; 3 Department of Obstetrics and Gynecology, Oulu University Hospital, Wellbeing Services County of North Ostrobothnia, 90220 Oulu, Finland; 4 Northern Finland Birth Cohort, Infrastructure for Population Studies, Faculty of Medicine, University of Oulu, 90014 Oulu, Finland; paula.pesonen@oulu.fi; 5 Research Unit of Population Health, University of Oulu, 90014 Oulu, Finland; sylvain.sebert@oulu.fi (S.S.); m.jarvelin@imperial.ac.uk (M.-R.J.); 6 Australian Centre for Precision Health, Cancer Research Institute, University of South Australia, Adelaide 5000, Australia; elina.hypponen@unisa.edu.au; 7 Unit of Clinical and Health Sciences, University of South Australia, Adelaide 5000, Australia; 8 South Australian Health and Medical Research Institute, Adelaide 5000, Australia; 9 Department of Epidemiology and Biostatistics, MRC Centre for Environment and Health, School of Public Health, Imperial College, London W2 1PG, UK; 10 Department of Life Sciences, College of Health and Life Sciences, Brunel University London Kingston Lane, Uxbridge UB8 3PH, UK; 11 Unit of Primary Care, Oulu University Hospital, Wellbeing Services County of North Ostrobothnia, 90220 Oulu, Finland; 12 Research Unit of Biomedicine and Internal Medicine, University of Oulu, 90014 Oulu, Finland; 13 Department of Gastroenterology and Metabolism, Poznan University of Medical Sciences, 61-701 Poznan, Poland; 14 Department of Pediatrics and Adolescence, Oulu University Hospital, Wellbeing Services County of North Ostrobothnia, 90220 Oulu, Finland

**Keywords:** 25-hydroxyvitamin D, fecundability, infertility, miscarriages, population-based study, reproduction, vitamin D

## Abstract

Background: Infertility and fecundability problems have been linked with lower 25-hydroxyvitamin D (25(OH)D) concentrations, but studies conducted with small, heterogenous or selected populations have shown inconsistent results. Methods: This study included women at age 31 from prospective population-based Northern Finland Birth Cohort 1966. Serum 25(OH)D concentrations were evaluated between women with or without previous infertility examinations or treatments (infertility group, *n* = 375, reference group, *n* = 2051) and time to pregnancy (TTP) of over 12 months (decreased fecundability group, *n* = 338) with a wide range of confounders. Furthermore, 25(OH)D concentrations were also compared among reproductive outcomes. Results: The mean 25(OH)D concentration was lower and 25(OH)D < 30 nmol/L was more frequent in women with a history of infertility compared to reference group. Moreover, 25(OH)D > 75 nmol/L was more frequent in the reference group. The mean 25(OH)D concentration was lower in women who had had multiple miscarriages. Both history of infertility (β = −2.7, 95% confidence interval (CI) −4.6, −0.7) and decreased fecundability associated with lower 25(OH)D concentration (β = −4.1, 95% CI −7.4, −0.8) after adjustments. In conclusion, this population-based study demonstrated that previous infertility and decreased fecundability were associated with lower 25(OH)D.

## 1. Introduction

Reproductive problems, such as infertility and prolonged time to pregnancy as well as insufficient vitamin D status, are common in Nordic countries [1,2,3]. Since vitamin D receptors and enzymes linked to vitamin D metabolism have been found in the pituitary gland, uterus, ovaries and placenta in women, a connection between vitamin D status and endocrinological and reproductive functions has been suggested [4,5].

Multiple conditions, including obesity and polycystic ovary syndrome, can influence decreased fertility, but lifestyle also interacts with fecundability [1]. Given the increasing number of patients suffering from infertility, it has become necessary to identify possible dietary effects [6]. The amount of solar vitamin D is reduced due to a long winter season in northern latitudes [7]. Thus, identifying impaired vitamin D status and ensuring sufficient vitamin D intake from the diet could be an easy way to improve fecundability [8]. Previous animal studies have suggested an inverse association between vitamin D status and infertility, but in human studies, the findings have been controversial [9,10]. The positive actions of vitamin D in fertility may arise from the endocrine pathways of reproduction [11]. Decreased serum 25-hydroxyvitamin D (25(OH)D) concentrations have been found in women during infertility treatments [8]. Promising results have been observed from studies investigating the impact of an adequate vitamin D status on improved rates of infertility treatments [12].

Studies investigating vitamin D status and pregnancy rates in healthy women without previous infertility problems have shown conflicting results, but they have mainly been conducted with small sample sizes, different age distributions, varying ethnic backgrounds or selected patient groups [13,14,15,16]. Large observational studies conducted in general populations are lacking. The earlier studies also excluded women with previous infertility or diseases and thus did not reflect the situation at the population level [17]. The aim of this study was to investigate if previous infertility problems, decreased fecundability, and impaired reproductive outcomes associated with 25(OH)D concentrations in women at age 31. We hypothesized that in women with earlier infertility vitamin D status might be lower compared to women without infertility.

## 2. Materials and Methods

### 2.1. Study Population

**Figure 1:**
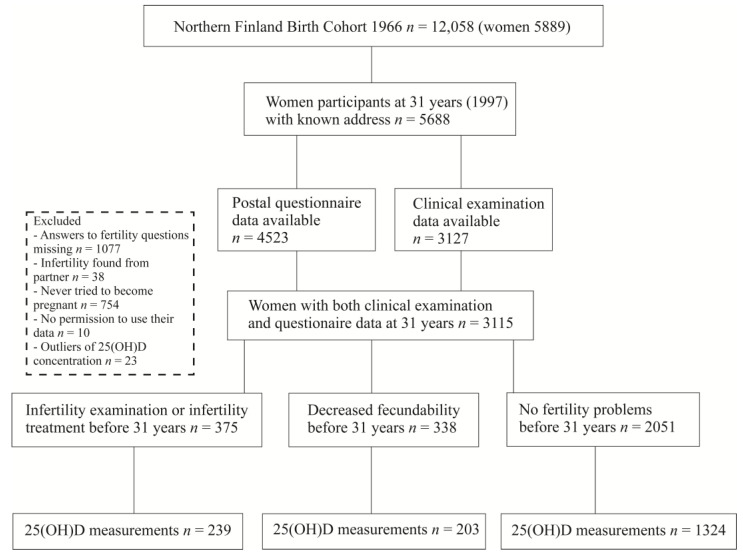
Flow chart showing the study population. 25(OH)D: 25-hydroxyvitamin D.

The study utilized a large prospective general population, the Northern Finland Birth Cohort 1966 (NFBC1966) [18]. The NFBC1966 has been previously described in detail [19]. Follow-ups were carried out on the cohort at ages 1, 14, 31 and 46. In this study, we used data from follow-up at age 31 (in 1997), which included a comprehensive questionnaire and a clinical examination with blood samples (Figure 1) [19].

The questionnaire administered when the cohort was aged 31 covered questions about family, social background, lifestyle, health and fertility and was sent to 11,543 (95%) participants whose addresses were known. Of these, 8690 (76.3%) answered, of whom 4523 were women. A request to undergo clinical examination at age 31 was sent simultaneously with the questionnaire, and 3127 women attended. Clinical examination data and postal questionnaire information were available from 3115 women [19].

The infertility population was formed from answers to the following questions: ‘Have you or has your partner been examined for infertility?’ and ‘Have you been treated for infertility?’ Women answering ‘Yes’ to either of the questions were placed in the ‘Infertility’ group (*n* = 375). Fecundability at 31 years was defined by time (in months) to the first pregnancy (TTP) from the time at which contraception was not used. Participants with active exposure to pregnancy for over one year were classified as having ‘decreased fecundability’ (*n* = 338). A total of 198 women were included in both infertility and decreased fecundability groups. The reference population included the women who had answered ‘No’ to both infertility questions and whose fecundability time was less than a year (*n* = 2051). Participants who had never tried to become pregnant (*n* = 754) or those with infertility problems due to their partner were excluded from the data (*n* = 38). Number of pregnancies, ectopic pregnancies, deliveries and miscarriages were categorized as zero, one and two or more from the answers to the questionnaire administered to the cohort at age 31.

### 2.2. 25-Hydroxyvitamin D Measurements

At age 31, clinical examination fasted blood samples were drawn between 8 and 11 am and stored at −70 °C. After the serum was thawed, 25-hydroxyvitamin D (25(OH)D) concentrations were measured with liquid chromatography–tandem mass spectrometry (LC-MS/MS; Elstree, Hertfordshire, UK). The measurement method has been explained in detail in a previous study [20]. To convert 25(OH)D measurements to the Vitamin D Standardization Program-calibrated concentrations to be comparable with other studies, a subset of 25(OH)D samples was later reanalyzed with a chemiluminescence microparticle immunoassay (CMIA) Architect i2000SR automatic analyzer (Abbott Diagnostics). The Center for Disease Control and Prevention’s Vitamin D Standardization-Certification Program (VDSP) has certified the assay [21]. 

Furthermore, 25(OH)D measurements and previous fertility information were available from a total of 1789 women. Observations lying abnormally far from other values were defined as outliers [22]. The interquartile range (IQR) was calculated to find extreme outliers of 25(OH)D measurements. Twenty-three outliers of 25(OH)D values were excluded using the first quartile cut-off, −1.5 × IQR for the lower limit, and the third quartile cut-off, +1.5 × IQR for the upper limit [22]. After excluding outliers, there were 239 women in the infertility group with 25(OH)D measurement, 203 women in the decreased fecundability group and 1324 in the reference group without fertility problems. We found no difference in the results between the full sample and after the exclusion of outliers. In addition, 25(OH)D concentrations were compared in clinical 25(OH)D status groups and in quartiles between the study groups. Moreover, 25(OH)D status groups were defined as <30, 30–50, 50–75 and >75 nmol/L based on Institute of Medicine and Endocrine Society guidelines [23,24].

### 2.3. Covariates

Background characteristics were determined based on the questionnaire. Relationship status was categorized as ‘ever been in a relationship (married, cohabiting, separated and widowed)’ or ‘no relationship’. Occupational status was classified as upper-level employees, lower-level employees/entrepreneurs, manual workers/farmers and non-workers. Alcohol consumption was defined as abstainer (0 g/d), low-risk drinking (≤20 g/d) and at-risk drinking (>20 g/d), estimated based on the intake of spirits, beer and wine in the last six months before the questionnaire [25]. Smoking was defined as non-smoker or current smoker. Physical activity (PA) was calculated as the metabolic equivalent of task (MET) scores in hours per week from the duration and frequency of leisure-time activities [26]. Information on vitamin D supplementation data have been represented previously [20]. In brief, vitamin D supplementation was asked in the postal questionnaire via the question ‘How often do you use the following medication? Vitamins or trace elements 1. Not at all, 2. Sometimes, 3. Regularly or continually’, including strength and dose. Participants who said they took any vitamin D-containing multivitamin or dietary supplementation were classified to ‘vitamin D supplementation user’ group. Participants without the use of vitamin D supplementation or vitamin D-containing multivitamins were categorized to ‘no vitamin D supplementation use’ group [20].

The season of blood sampling was categorized as low vitamin D season (November–May) and high vitamin D season (June–October) [27]. Information from the Finnish Population Register Centre was utilized to determine the latitude of participants’ residence, divided into three groups: 60° N (Helsinki and other provinces in middle and southern Finland), 65° N (the city of Oulu) and ≥65° N (the northernmost provinces of Oulu and Lapland) [28]. BMI (kg/m^2^) was calculated based on participants’ weight (kg) and height (cm), which were measured during clinical examination by well-trained nurses. If clinical information was missing, the answer was obtained from the postal questionnaire. Clinically measured and self-reported BMI gave similar results [29].

### 2.4. Statistical Analysis

Histogram normality curves were utilized to evaluate the distribution of continuous variables. We used an independent sample *t*-test or one-way analysis of variance when appropriate for the normally distributed continuous variables (BMI and 25(OH)D). The Mann–Whitney U test was used for the variable PA because of skewed distribution. Pearson’s chi-square test was utilized for categorical variables. Results are presented as mean (SD), median [IQR] and prevalence (%).

Multivariable linear regression models were used to determine independent association between a history of infertility and 25(OH)D concentration, and between a history of decreased fecundability and 25(OH)D concentration. 25(OH)D was the dependent variable in all the models. Infertility or decreased fecundability Model 1 was adjusted with season of the blood sampling, latitude and laboratory batch effect. Fully adjusted infertility or decreased fecundability Model 2 included season of the blood sampling, latitude, laboratory batch effect, relationship status, BMI and PA as independent variables. Infertility Model 2 also included alcohol consumption and smoking. Confounding factors in the models were selected based on statistically significant results from the abovementioned tests and previous literature [28,30]. After the variables were tested in the models one by one, they were all added in the regression models simultaneously. We investigated two-way interactions between the main explanatory variables in all the above-mentioned models. Only significant interaction terms of the independent variables were included in the final models based on goodness-of-fit tests.

As a sensitivity analysis, we performed one multivariable linear regression model including vitamin D supplementation use (Supplementary Tables S1 and S2A) and another in which we excluded women who were pregnant at the age 31 follow-up (Supplementary Tables S1 and S2B), since hemodilution might cause alteration in 25(OH)D concentrations [31]. The pregnant women’s weeks of gestation were not known.

**Figure 2:**
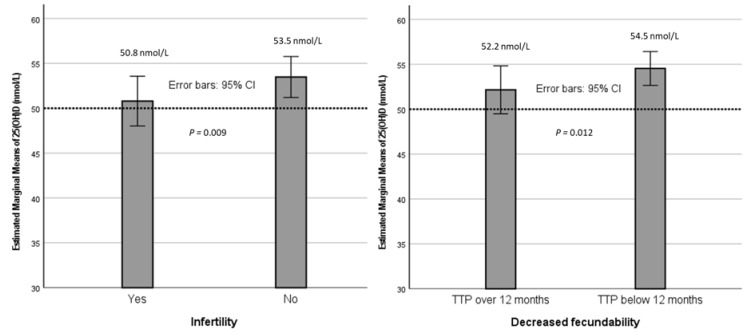
Adjusted mean 25(OH)D concentrations in women with a history of infertility and reference group, and in women with decreased fecundability and reference group. 25(OH)D: 25-hydroxyvitamin D, TTP: Time to first pregnancy.

*p* value of below 0.05 was considered statistically significant. The statistical analyses were executed using IBM SPSS Statistics for Windows, Version 26 (IBM Corp, Armonk, NY, USA) and RStudio Version 1.1.456 (https://www.rstudio.org accessed on 10 June 2021). Figure 1 was created using CorelDRAW Graphics Suite 2019, Version 21.0.0.593 (Corel Corporation, Ottawa, ON, Canada). Figure 2 was conducted with IBM SPSS Statistics for Windows, Version 26 (IBM Corp, Armonk, NY, USA).

The ethical committee of the Northern Ostrobothnia Hospital District and the University of Oulu originally approved the cohort study (94/2011, 12/2003). The methods followed the 1964 Declaration of Helsinki and its later amendments. All participants have given written informed consent to use their cohort data. It is not possible to identify the study participants.

## 3. Results

### 3.1. Background Characteristics

Table 1 presents the background characteristics of the study participants. 

Women with decreased fecundability had been in relationships more frequently than reference women (*p* = 0.04). Women with infertility had more frequent alcohol consumption at ‘at-risk’ levels and they were more often current smokers than those in the reference group (*p* = 0.04 and *p* = 0.03, respectively). In both infertility and decreased fecundability groups, women had a higher BMI than the reference women (*p* = 0.02 and *p* = 0.03, respectively). The amount of PA was higher in women with decreased fecundability (*p* = 0.02) than in the reference group. The other background characteristics, vitamin D supplementation use, dose of vitamin D supplementation or pregnancies during the follow-up did not differ between the study groups (Table 1, Supplementary Table S1).

### 3.2. Vitamin D Status in the Study Population

In women with previous infertility, unadjusted mean serum 25(OH)D concentration was lower (51.2 nmol/L, SD 18.9, *p* = 0.019), but no significant difference was found between the decreased fecundability group (53.1 nmol/L, SD 17.9, *p* = 0.39) and the reference group (54.2 nmol/L, SD 18.2, Table 2). In addition, serum 25(OH)D concentration <30 nmol/L was more frequent in women with a history of infertility compared to women without fertility problems, whereas a serum 25(OH)D concentration >75 nmol/L was more frequent in women with normal fecundability (*p* = 0.019). These findings did not occur in quartiles or in women with decreased fecundability (Table 2).

### 3.3. Vitamin D Status in Different Reproductive Outcome Groups

Table 3 presents unadjusted mean serum 25(OH)D concentrations and distributions of vitamin D groups in relation to different reproductive outcomes. 

There were no differences between the mean 25(OH)D concentrations or vitamin D status groups and occurrence of preceding pregnancies, ectopic pregnancies or deliveries. However, in women who had had two or more miscarriages, the mean 25(OH)D concentrations were lower (*p* = 0.04). Moreover, multiple miscarriages were more prevalent in the women with 25(OH)D concentration <30 nmol/L and less frequent in the group with concentration >75 nmol/L than the other groups, but the difference was not significant (*p* = 0.079).

### 3.4. Association of Impaired Fertility and 25(OH)D Concentration

In adjusted multivariable linear regression infertility Model 1, a significant negative association was observed between a history of infertility and 25(OH)D concentrations (β = −2.3, 95% confidence interval (CI) −4.2, −0.3, *p* = 0.022, R^2^ = 0.44, Table 4). In the other adjusted Model 1, a history of decreased fecundability had no significant association with 25(OH)D concentration (β = −1.9, 95% CI −4.0, 0.1, *p* = 0.066, R^2^ = 0.41, Table 4). However, in the fully adjusted Model 2, both a history of infertility (β = −2.6, 95% CI −4.6, −0.7, *p* = 0.009, R^2^ = 0.44) and decreased fecundability (β = −4.2, 95% CI −7.5, −1.0, *p* = 0.012, R^2^ = 0.43) associated with lower 25(OH)D concentration.

Figure 2 is showing the differences in adjusted mean 25(OH)D concentrations between women with earlier infertility and reference groups and between decreased fecundability and reference groups. The estimated marginal means of 25(OH)D serum concentrations were 50.8 nmol/L and 53.5 nmol/L in infertility vs. reference groups (*p* = 0.009), and 52.2 nmol/L and 54.5 nmol/L in the decreased fecundability vs. reference groups (*p* = 0.012), respectively.

## 4. Discussion

In our analyses of a large comprehensive population-based birth cohort, previous infertility and decreased fecundability were associated with lower 25(OH)D concentrations in women in their early 30s. In women who had had two or more miscarriages, the mean 25(OH)D concentrations were lower compared to women who had had one miscarriage or no miscarriages.

Few studies have assessed the association between infertility problems and vitamin D status in women or TTP in a cohort study setting in a general population. Previously, Fung et al. showed that women with 25(OH)D concentrations over 50 nmol/L had a higher possibility of pregnancy than those with 25(OH)D concentrations below 50 nmol/L [13]. Similar results have been found in studies from the US, where 25(OH)D concentrations have been associated positively with a probability of pregnancy [15,17]. However, opposite results were observed in Danish and Italian studies, but the number of participants in these studies was low and the time of follow-up was short [14,16]. Møller et al. found no associations between vitamin D status and fertility or pregnancy outcomes [14]. In that study, the study population included only healthy women who used oral contraceptives and controls were included only in winter time [14]. Somigliana et al. measured 25(OH)D in already pregnant women, which might have affected the 25(OH)D concentrations [16]. All but one of these studies focused mainly on healthy women and excluded those with a history of infertility problems [13,14,16,17]. One study also lacked information about vitamin D supplementation use [15]. In these studies, the definition of 25(OH)D deficiency varied; in studies from Denmark and US, the deficiency was defined as 50 nmol/L [14,15,17] whereas in a study from Italy, the threshold of insufficiency was set at 50 nmol/L and deficiency at 25 nmol/L [16]. However, our study did not aim to define the limit of deficiency for 25(OH)D concentration in women with infertility problems. Supplementary Table S3 represents studies from the last five years evaluating the association of fertility and vitamin D.

Vitamin D has been suggested to have immunomodulatory effects [32,33], which may contribute to embryonic implantation, placentation and pregnancy success [33,34]. In our study population, the mean 25(OH)D concentration was lower in women who had had two or more miscarriages than in those who had had one miscarriage or no miscarriages. Previous results have been inconsistent on a possible association between miscarriages and 25(OH)D [15,35,36,37,38], but the problem in these observations was that study groups were small, and the results should therefore be interpreted with caution. Still, future research is warranted to evaluate the connection of miscarriages and 25(OH)D.

Steroidogenesis of sex hormones is regulated by multiple enzymes [39]. Previously, it has been proposed that vitamin D positively influences fertility by affecting the synthesis of these enzymes, such as 17β-hydroxysteroid dehydrogenase and aromatase [40,41]. 17β-hydroxysteroid dehydrogenase affects fertility by regulating the concentration of sex steroid hormones [40] and aromatase by catalyzing the synthesis of estrogen [41]. Furthermore, a positive correlation of 25(OH)D concentrations and ovarian reserve marker anti-Müllerian hormone levels has been found, suggesting that vitamin D could improve folliculogenesis [11,42]. The relevance of this association to fertility requires further investigation.

The interplay between lifestyle factors (BMI, smoking and alcohol consumption), vitamin D and infertility is a complex entity and causality is hard to establish. High BMI has been associated with a decreased bioavailability of vitamin D [43], lower live birth rates after IVF treatment [44] and with increased risk of miscarriage [45]. Smoking impairs fertility by several mechanisms [46,47] and is associated with lower vitamin D levels in both pregnant and non-pregnant women [48,49]. The results of studies on alcohol consumption and infertility are controversial [47], and the association of alcohol consumption with vitamin D status remains inconclusive [50].

In addition, a promising positive role of vitamin D regarding the success of infertility treatments has been postulated in some studies [8,12]. Adequate vitamin D status might increase the probability of pregnancy and live births in women during infertility treatments, such as in vitro fertilization (IVF) [12]. Vitamin D supplementation was found to alter gene expression in granulosa cells in women undergoing IVF [51]. However, contrasting observations have also been presented in some studies [52,53]. In women treated with IVF or intracytoplasmic sperm injection, 25(OH)D concentrations were positively associated with fertilization rates, but no relation was demonstrated with clinical pregnancy or live birth rates [54]. Similarly, 25(OH)D concentrations did not differ among number of pregnancies or deliveries in our study population. However, although 25(OH)D concentrations were higher in the reference population, the mean was still within the 50 nmol/L insufficiency limit and the difference between the groups was moderate. Furthermore, 25(OH)D concentrations over 50 nmol/L have been shown to inhibit parathormone elevation and prevent rickets [23]. The beneficial health effects which improve fertility might require even higher concentrations, but the exact cut-off is still unknown and requires further assessments.

Our study has multiple strengths. Due to our comprehensive general population-based cohort study setting, we were able to evaluate and adjust the results by using a wide range of relevant confounding factors in our homogenous population. Our study included women of the same age and ethnic background, both of which affect vitamin D status. We were able to utilize information about vitamin D supplementation and multivitamin use. The participation rate in our study was relatively high. We used VDSP-calibrated 25(OH)D concentrations, which makes the results comparable with other vitamin D studies [21]. Fertility populations were characterized from multiple angles by using infertility examinations and treatments, fecundability and reproductive outcomes [55].

Our study also has limitations. It was based on observational birth cohort data, and causality cannot be assessed. Moreover, one single 25(OH)D measurement was available in the study, and we did not have 25(OH)D concentrations measured before the onset of fertility problems. However, previous studies have noted that individual 25(OH)D concentrations might predict 25(OH)D concentration later in life [56,57]. The confounding factors might have altered the timing of infertility. Information for this study was collected at age 31, but in Finland, the mean age of first delivery in 1997 was 27.7 and the study captured the period that was relevant for an investigation of early fertility problems [58]. The lack of precise dietary data is an acceptable limitation, since the national fortification of dairy products and fat spreads with vitamin D was launched after the 31-year follow-up in 2002 [28].

## 5. Conclusions

In conclusion, our population-based birth cohort study showed an association with an increased rate of previous infertility problems and decreased fecundability in women with lower 25(OH)D concentrations, albeit the difference in mean vitamin D status was moderate. The result provides new information on the patient group that might be at increased risk for vitamin D insufficiency. In the future, studies are required to assess the consequences of this observation.

## Figures and Tables

**Table 1:** Background characteristics of study participants at age 31.

	Infertility * (*n* = 239)	Decreased Fecundability ** (*n* = 203)	No Fertility Problems (*n* = 1324)	*p* Value *	*p* Value **
Relationship status, *n* (%)				0.94	0.04
Ever been in relationship ^a^	225 (95.3)	198 (98.5)	1261 (95.5)		
No relationship	11 (4.7)	3 (1.5)	60 (4.5)		
Occupational status, *n* (%)				0.64	0.09
Higher-level employee	41 (17.4)	40 (20.0)	186 (14.7)		
Lower-level employee/ entrepreneur	111 (47.0)	107 (53.5)	646 (51.1)		
Manual worker/farmer	48 (20.3)	30 (15.0)	251 (19.8)		
Not working	36 (15.3)	23 (11.5)	182 (14.4)		
Latitude ^b^, *n* (%)				0.996	0.06
60° N	30 (12.6)	22 (10.8)	168 (12.7)		
65° N	44 (18.4)	51 (25.2)	241 (18.2)		
>65° N	165 (69.0)	130 (64.0)	915 (69.1)		
Season ^c^, *n* (%)				0.17	0.77
High vitamin D season	143 (60.3)	134 (66.0)	856 (64.9)		
Low vitamin D season	94 (39.7)	69 (34.0)	462 (35.1)		
Alcohol consumption ^d^, *n* (%)				0.02	0.10
Abstainer	23 (9.9)	20 (10.1)	150 (11.6)		
Low-risk drinker	199 (85.0)	170 (85.4)	1119 (86.3)		
At-risk drinker	12 (5.1)	9 (4.5)	27 (2.1)		
Smoking, *n* (%)				0.03	0.15
Non-smoker	170 (71.4)	148 (73.3)	1027 (77.9)		
Current smoker	68 (28.6)	54 (26.7)	292 (22.1)		
Physical activity ^e^ (MET/h), median [IQR]	11.8 [16.8]	13.1 [17.3]	11.3 [16.9]	0.14	0.03
BMI (kg/m^2^), mean (SD)	25.0 (5.6)	25.0 (5.3)	24.0 (4.4)	0.017	0.03

* *p* value between women with previous infertility examination or infertility treatment vs. women without fertility problems. ** *p* value between women with decreased fecundability vs. women without fertility problems. Categories compared with Pearson’s chi-square test, medians with Mann–Whitney test and means with independent samples *t*-test. ^a^ Ever been married, cohabiting, separated and widowed. ^b^ 60° N: Helsinki and surrounding areas. 65° N: the city of Oulu. >65° N: the northernmost provinces of Oulu and Lapland. ^c^ High vitamin D season: 1 June–31 October (summer and autumn). Low vitamin D season: 1 November–31 May (winter and spring). ^d^ Abstainer (0 g/d), low-risk drinker (≤20 g/d), and high-risk drinker (>20 g/d). ^e^ MET scores: physical activity in hours per week (frequency and duration of leisure-time activities). IQR: interquartile range, MET: metabolic equivalent of task, °N: north latitude, SD: standard deviation.

**Table 2:** Serum 25-hydroxyvitamin D (25OHD) concentrations and distributions in study population aged 31.

	Infertility * (*n* = 239)	Decreased Fecundability ** (*n* = 203)	No Fertility Problems (*n* = 1324)	*p* Value *	*p* Value **
25(OH)D (nmol/L), mean (SD)	51.2 (18.9)	53.1 (17.9)	54.2 (18.2)	0.019	0.39
25(OH)D groups, *n* (%)				0.019	0.86
<30	38 (15.9)	20 (9.9)	123 (9.3)		
30–50	77 (32.2)	72 (35.5)	439 (33.2)		
50–75	97 (40.6)	83 (40.9)	584 (44.1)		
>75	27 (11.3)	28 (13.7)	178 (13.4)		
25(OH)D quartiles, *n* (%)				0.063	0.46
<39.1	71 (29.7)	54 (26.6)	317 (23.9)		
39.1–53.1	59 (24.7)	44 (21.7)	327 (24.7)		
53.1–66.5	63 (26.4)	57 (28.1)	327 (24.7)		
>66.5	46 (19.2)	48 (23.6)	353 (26.7)		

* *p* value between women with previous infertility examination or infertility treatment vs. women without fertility problems. ** *p* value between women with decreased fecundability vs. women without fertility problems. Means compared with independent samples *t*-test and categories with Pearson’s chi-square test. 25(OH)D: 25-hydroxyvitamin D, SD: standard deviation.

**Table 3:** Mean 25-hydroxyvitamin D (25OHD) concentrations and distributions at age 31 between reproductive characteristics.

	25(OH)D, nmol/L	*p* Value *	25(OH)D Groups, *n* (%)				*p* Value **
	Mean (SD)		<30 nmol/L	30–50 nmol/L	50–75 nmol/L	>75 nmol/L	
Pregnancies		0.38					0.42
0 (*n* = 229)	52.9 (18.1)		20 (8.5)	85 (10.8)	100 (10.0)	24 (8.0)	
1 (*n* = 486)	54.6 (17.9)		43 (18.3)	153 (19.5)	226 (22.5)	64 (21.5)	
≥2 (*n* = 1607)	53.5 (18.5)		172 (73.2)	548 (69.7)	677 (67.5)	210 (70.5)	
Miscarriages		0.04					0.08
0 (*n* = 1778)	54.2 (18.1)		164 (75.9)	596 (82.2)	789 (84.3)	229 (83.0)	
1 (*n* = 296)	51.7 (18.6)		38 (17.6)	105 (14.5)	118 (12.6)	35 (12.7)	
≥2 (*n* = 79)	51.0 (19.5)		14 (6.5)	24 (3.3)	29 (3.1)	12 (4.3)	
Ectopic pregnancies		0.51					0.7
0 (*n* = 2047)	53.9 (18.3)		201 (97.1)	689 (97.5)	891 (97.0)	266 (96.0)	
≥1 (*n* = 63)	55.5 (17.4)		6 (2.9)	18 (2.5)	28 (3.0)	11 (4.0)	
Deliveries		0.07					0.08
0 (*n* = 290)	55.9 (18.0)		17 (7.8)	99 (13.4)	128 (13.5)	46 (15.9)	
1 (*n* = 591)	53.9 (17.9)		56 (25.7)	189 (25.6)	272 (28.7)	74 (12.5)	
≥2 (*n* = 1306)	53.2 (18.5)		145 (66.5)	450 (61.0)	549 (57.9)	162 (57.4)	

* *p* value from one-way analysis of variance. ** *p* value from Pearson’s chi-square test. 25(OH)D: 25-hydroxyvitamin D, SD: standard deviation.

**Table 4:** Association of 25-hydroxyvitamin D and relevant exposures in multivariable linear regression models in women with previous infertility or decreased fecundability.

Multivariable Linear Regression Models	β Coefficient	95% CI of β	*p* Value
Infertility Model 1 ^a,b^	−2.3	−4.2, −0.3	0.022
Infertility fully adjusted Model 2 ^b,c^	−2.6	−4.6, −0.7	0.009
Decreased fecundability Model 1 ^b,d^	−1.9	−4.0, 0.1	0.066
Decreased fecundability fully adjusted Model 2 ^b,e^	−4.2	−7.5, −1.0	0.012

^a^ Infertility Model 1 also included latitude, season and laboratory effect, R^2^ = 0.44. No significant interactions in the model. ^b^ No fertility problems as reference category. ^c^ Infertility Model 2 also included relationship status, latitude, season, alcohol consumption, smoking, laboratory effect, BMI and physical activity, R^2^ = 0.44. Interactions in the final model: Latitude × BMI, latitude × physical activity, smoking × sample season. ^d^ Decreased fecundability Model 1 also included relationship status, latitude, season and laboratory effect, R^2^ = 0.41. No significant interactions in the model. ^e^ Decreased fecundability Model 2 also included latitude, season, relationship status, laboratory effect, BMI and physical activity, R^2^ = 0.43. Interactions in the final model: Latitude × BMI, latitude × physical activity, decreased fecundability × physical activity. CI: Confidence interval.

## Data Availability

NFBC1966 data is available from the University of Oulu, Infrastructure for Population Studies. Permission to use the data can be applied for research purposes via an electronic material request portal. In the use of data, we follow the EU general data protection regulation (679/2016) and Finnish Data Protection Act. The use of personal data is based on cohort participant’s written informed consent at his/her latest follow-up study, which may cause limitations to its use. Please contact NFBC project center (NFBCprojectcenter@oulu.fi) and visit the cohort website (https://www.oulu.fi/en/university/faculties-and-units/faculty-medicine/northern-finland-birth-cohorts-and-arctic-biobank accessed on 18 March 2019) for more information.

## Supplementary Materials

The following supporting information can be downloaded at: https://www.mdpi.com/article/10.3390/nu15112522/s1, Table S1: Vitamin D supplementation use and pregnancy information during the follow-up visit in the study population at 31 years of age. Table S2A: Association of 25-hydroxyvitamin D and relevant exposures in multivariable linear regression model as a sensitivity analysis including vitamin D supplementation use. Table S2B: Association of 25-hydroxyvitamin D and relevant exposures in multivariable linear regression Models 5 and 6 as a sensitivity analysis excluding pregnant women. Table S3: Studies from the last five years evaluating the association of fertility and vitamin D.
