# Supplementary material for: Vitamin D Status in Women with a History of Infertility and Decreased Fecundability: A Population-Based Study
Source: Nutrients. 2023 May 29;15(11):2522. doi: 10.3390/nu15112522 (PMC10255371; doi:10.3390/nu15112522)
Supplement: Supplementary file 1 [file nutrients-15-02522-s001.zip › Supplementary_Table S1.pdf]

Supplementary material S1

Supplementary Table S1. Vitamin D supplementation use and pregnancy information during the follow-up visit in the study population at 31 years of age.

	**Infertility* (n = 239)**	**Decreased fecundability** (n = 203)**	**No fertility problems (n = 1324)**	***p* value***	***p* value****
Vitamin D supplementation, n (%)				0.78	0.18
Yes	14 (5.9)	8 (3.9)	84 (6.3)		
No	225 (94.1)	195 (96.1)	1240 (93.7)		
Vitamin D supplementation dose, (μg/day), median [IQR]	10.0 [5.2]	10.0 [5.0]	10.0 [2.5]	0.60	0.34
Pregnancy during the follow-up visit, n (%) [a]				0.96	0.24
Yes	23 (9.9)	25 (12.4)	127 (9.8)		
No	210 (90.1)	176 (87.6)	1174 (90.2)		

* *p* value comparing women with previous infertility examination or infertility treatment vs women without fertility problems. ** *p* value comparing women with decreased fecundability vs women without fertility problems. Categories compared with Pearson's chi-square test and medians with Mann–Whitney U test. [a] Weeks of gestation not known. SD: standard deviation.
