# Supplementary material for: Vitamin D Status in Women with a History of Infertility and Decreased Fecundability: A Population-Based Study
Source: Nutrients. 2023 May 29;15(11):2522. doi: 10.3390/nu15112522 (PMC10255371; doi:10.3390/nu15112522)
Supplement: Supplementary file 1 [file nutrients-15-02522-s001.zip › Supplementary_Table S2.pdf]

Supplementary material S2

Supplementary Table S2A. Association of 25-hydroxyvitamin D and relevant exposures in multivariable linear regression model as a sensitivity analysis including vitamin D supplementation use.

Multivariable linear regression Models	β Coefficient	95 % CI of β	*p* value
Infertility fully adjusted Model [a,b]	-2.6	-4.6 to -0.6	0.009
Decreased fecundability fully adjusted Model [b,c]	-3.9	-7.2 to -0.7	0.019

[a] Model also included relationship status, vitamin D supplementation use, alcohol consumption, smoking, latitude, laboratory effect, BMI, and physical activity. For Model, R^2=0.45. [b] No fertility problems as reference category. [c] Model also included relationship status, vitamin D supplementation use, latitude, laboratory effect, BMI, and physical activity. For Model, R^2=0.44. CI: confidence interval.

Supplementary Table S2B. Association of 25-hydroxyvitamin D and relevant exposures in multivariable linear regression Models 5 and 6 as a sensitivity analysis excluding pregnant women.

Multivariable linear regression Models	β Coefficient	95 % CI of β	*p* value
Infertility fully adjusted Model [a,b]	-2.4	-4.5 to -0.3	0.026
Decreased fecundability fully adjusted Model [b,c]	-4.6	-8.0 to -1.2	0.008

[a] Model also included relationship status, alcohol consumption, smoking, BMI, physical activity, latitude, and laboratory effect. For Model, R^2=0.47. [b] No fertility problems as reference category. [c] Model also included relationship status, BMI, latitude and laboratory effect without significant association with 25(OH)D concentrations. For Model, R^2=0.45. CI: confidence interval.
