# Supplementary material for: Vitamin D Status in Women with a History of Infertility and Decreased Fecundability: A Population-Based Study
Source: Nutrients. 2023 May 29;15(11):2522. doi: 10.3390/nu15112522 (PMC10255371; doi:10.3390/nu15112522)
Supplement: Supplementary file 1 [file nutrients-15-02522-s001.zip › Supplementary_Table S3.pdf]

Supplementary material S3

Supplementary Table S3. Studies from the last five years evaluating the association of fertility and vitamin D.

Study	Design	Aim	Methods and background characteristics (BC)	Reference range of 25(OH)D (nmol/L)	Results
Mumford et al 2018, Lancet Diabetes Endocrinology	Secondary analysis of a prospective cohort	Determine preconception vitamin D and pregnancy outcomes among women with proven fecundity	- 1191, 18–40-year-old women attempting pregnancy with one to two prior miscarriages<br>- 25(OH)D measurements at preconception (baseline) and 8 weeks gestation<br>- Relative risk for live birth, pregnancy and miscarriage, and fecundability odds ratios<br>- BC: Age, education, BMI, race, alcohol consumption, PA, season, treatment assignment, vitamin use, number of previous losses, and number of prior live births	- Deficient 25(OH)D <50<br>- Insufficient 25(OH)D <75 nmol/L<br>- Sufficient 25(OH)D ≥75	- 46.6% had vitamin D concentrations ≥75 nmol/L and were more likely to achieve clinical pregnancy and live birth than women with 25(OH)D <75 nmol/L.<br>- Among women who achieved pregnancy, preconception 25(OH)D, but not 8 weeks gestation, was associated with lower risk of pregnancy loss<br>- No association was observed with fecundability
Jukic et al 2019, Human reproduction	Prospective cohort study	Assess the association of preconception 25(OH)D and fecundability	- 522 women attempting to become pregnant between 2010 and 2016<br>- Women completed online daily and monthly diaries until a positive home pregnancy test or 12 months elapsed<br>- 25(OH)D measurements at the study entry<br>- BC: age, race, education, BMI, gravidity, time since estrogen use, poorly timed intercourse,	- High 25(OH)D at least 125<br>- Insufficient 25(OH)D 50–75<br>- Deficient 25(OH)D <50<br><br>- 25(OH)D categorized as <50, 50–75, 75–	- 257 conceived during the study.<br>- Mean 25(OH)D was 90 nmol/L.<br>- 10% higher fecundability with each 25nmol/L increase in 25(OH)D<br>- Suggestive dose-response association with 25(OH)D when comparing the lowest and the highest categories of 25(OH)D<br>- Compared to women with 25(OH)D of 75-100 nmol/L, women below 50nmol/L had 45% reduction in fecundability and

			attempted cycles at blood draw, alcohol and caffeine consumption, PA	100, 100–125, >125	women with at least 125 nmol/L had 35% increase in fecundability<br>- Probability of taking longer than six months to conceive was 51% in 50nmol/L, 28% in 75-100 nmol/L and 15% in 125 nmol/L.
Zhang et al, 2022, Nutrients	Prospective cohort study	Evaluate the effect of serum 25(OH)D, among preconception, on fecundity, and the associations between 25(OH)D concentrations preconception, during pregnancy and pregnancy outcomes	- 200 preconception couples attempting to conceive and followed-up until childbirth.<br>- Time to pregnancy was collected via phone calls every two months or via a questionnaire during pregnancy<br>- 25(OH)D measurements from both partners at enrollment and from women during the second and third trimester of pregnancy<br>- BC: age, BMI, education, gravidity, smoking, alcohol consumption, multivitamin, calcium, folic acid use, milk, fish, liver intake, season	Sufficient 25(OH)D >75<br>- Insufficient 25(OH)D <75	- Couples had higher conception rates within six and shorter time to pregnancy if male partners had sufficient 25(OH)D compared with insufficient 25(OH)D.<br>- Compared to pregnant women with insufficient 25(OH)D in the third trimester of pregnancy, sufficient 25(OH)D was associated with reduced odds of anemia, longer gestational age and newborns' higher ponderal index
Subramanian et al, 2022, Human reproduction	Prospective time-to-pregnancy study	Assess the association of preconception vitamin D status and the risk of miscarriage	- 362 women aged 30-44 years, who were trying to conceive naturally in 2008–2015.<br>- 25(OH)D measurements at preconception<br>- Gestational age at pregnancy loss, time to miscarriage<br>- BC: age, race, BMI, education, exercise, alcohol and caffeine intake	<75, 75-100, >100 nmol/L<br><br>25(OH)D categorized: <75, 75–100 and ≥100. Collapsed the lowest two categories (<50 and 50–75)	- Hazard ratio for those with a low 25(OH)D level (<75 nmol/L) was 1.10 (CI: 0.62, 1.91) compared to the referent group (75–100 nmol/L). Among participants with a higher 25(OH)D level (≥100 nmol/L), the HR was 1.07 (CI: 0.62, 1.84).<br>- Findings of the study did not support an association between preconception vitamin D and miscarriage

25(OH)D: 25-hydroxyvitamin D, BC: Background characteristics, CI: Confidence interval, HR: Hazard ratio, PA: Physical activity.
